# *Dirofilaria repens* Infection Mimicking Lung Melanoma Metastasis

**DOI:** 10.1093/ofid/ofz049

**Published:** 2019-02-09

**Authors:** A Oliva, S Gabrielli, A Pernazza, A Pagini, T Daralioti, S Mantovani, S Mattiucci, G D’Amati, C M Mastroianni

**Affiliations:** 1 Department of Public Health and Infectious Diseases, Sapienza University of Rome, Rome, Italy; 2 Department of Radiological, Oncological and Pathological Sciences, Sapienza University of Rome, Rome, Italy; 3 Department of Thoracic Surgery, Sapienza University of Rome, Rome, Italy

**Keywords:** differential diagnosis, dirofilariasis, *Dirofilaria repens*, lung nodules, parasites

## Abstract

We describe a rare case of *Dirofilaria repens* infection presenting as peripheral lung nodules and mimicking a metastatic focus from a previously diagnosed cutaneous melanoma. To avoid invasive investigations before arriving at the correct diagnosis, dirofilariasis should be included as a part of the diagnostic process in subjects with lung nodules who live in (or have traveled to) endemic regions.

## CASE

**Figure 1.**
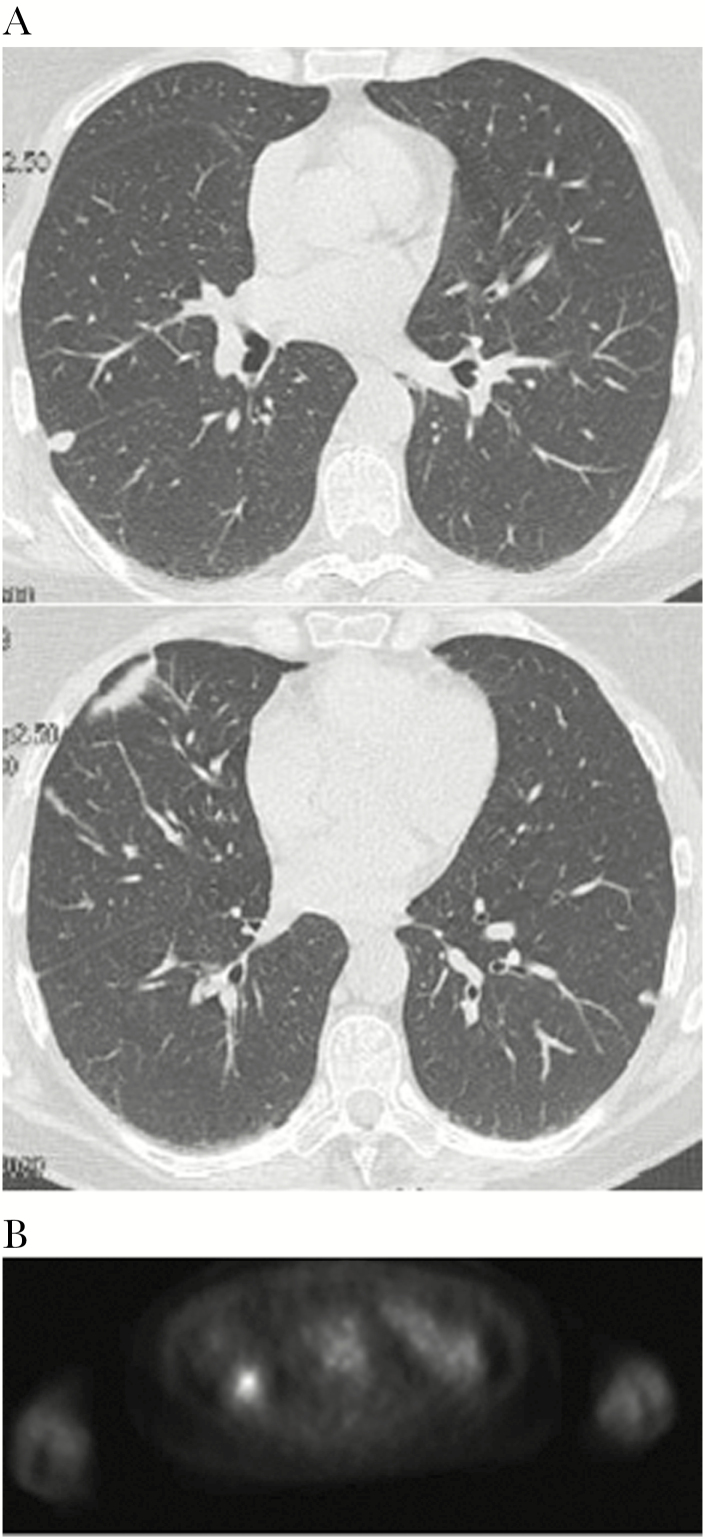
A computed tomography (CT) scan showed multiple nodules in the right lung without lymph node involvement, and the biggest (12 × 5 mm) was in the right lower lobe (RLL) (A). Fluorodeoxyglucose-positron emission tomography/CT confirmed the presence of multiples nodules, showing a low uptake only by the nodule in the RLL (1.7 SUV max) and no uptake in the rest of body (B).

A 55-year old Italian woman with a history of cutaneous melanoma was admitted to the Thoracic Surgery Unit of Policlinico Umberto I due to the appearance of pulmonary nodules on a positron emission tomography/computed tomography follow-up exam (Figure 1A, B).

**Figure 2.**
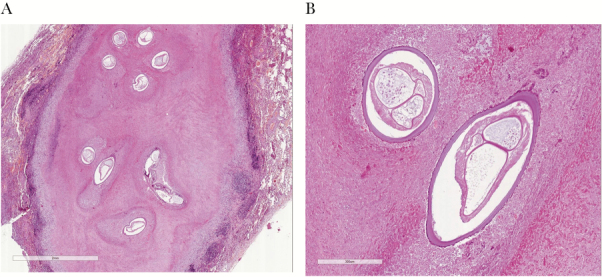
Pulmonary nodule filled with necrotic material and worms, surrounded by inflammatory cells. Hematoxylin and eosin (H&E). Original magnification 2x (A). High-power view of worms with features consistent with *Dirofilaria repens*; note the thick cuticle and the presence of internal organs. H&E. Original magnification 10x (B).

Physical examination and serum chemistry were normal. She was afebrile and in good condition. Due to the high suspicion of lung metastasis from the previously diagnosed melanoma, she underwent wedge resection in the right lower lobe. Gross examination of the surgical specimen revealed a nodular lesion of soft consistency with areas of necrosis, and histology confirmed extensive necrosis surrounded by chronic inflammatory reaction. The main finding consisted of the presence of worms embedded in the necrotic material, showing a thick cuticle and internal organs and exhibiting morphological features of a filarioid parasite (Figure 2A, B). For identification to the species level, polymerase chain reaction–DNA was performed. DNA was extracted from the paraffin block, and the mtDNA *cox1* gene fragment (about 650 bp) was amplified using filarioid-generic primers [1]. Sequence analysis showed a 100% match with a sequence of the same gene of the species *Dirofilaria repens*, deposited in GenBank (Accession Number DQ358814.1).

The patient had been born and was living in an urban area of Central Italy (Civitavecchia). Questioned on her habits, she reported recent travels to Northeast Italy and every year, in August, to a rural area in Bosnia Herzegovina. During these periodic trips, she recalled being frequently bitten by mosquitoes. Furthermore, the patient’s dog was analyzed by a veterinarian, and neither *Dirofilaria* nor any other parasite was found in the blood.

The patient was not given anti-infective therapy. At 3-month follow-up, physical examination and blood analyses were normal.

## DISCUSSION

Dirofilariasis is a mosquito-borne parasitosis caused by *Dirofilaria immitis* and *D. repens.* The first has a worldwide distribution, and it mainly causes benign pulmonary nodules. *D. repens* is limited to the Old World, causing subcutaneous nodules and intraocular infections [2].

These parasites circulate naturally between mosquitoes and canids, with humans as accidental hosts. *Dirofilaria* spp. is being increasingly detected in subcutaneous and/or ocular tissues and in asymptomatic, abortive internal sites of infection, that is, pulmonary nodules, with the clinical implication that these lesions are initially misidentified as malignancies, thus requiring invasive investigations before the correct diagnosis, which is mainly made during histopathology [2].

In the present case, the diagnosis was made after surgical excision of a nodule suspected for being lung metastasis, occurring several years after the melanoma finding. As for anamnesis, the patient reported several travels to endemic areas, including Northeast Italy and Eastern Europe, with frequent mosquito bites. It is noteworthy to underline that pulmonary nodules represent an unusual manifestation of a *D. repens* human infection, as they are typically caused by *D. immitis*.

Despite increasing reports of human dirofilariasis in Italy [3] and other European countries [4, 5], there is still a lack of awareness by clinicians, as the disease occurs mostly asymptomatically and accurate diagnostic tests are applied only in a few specialized laboratories. Thus, dirofilariasis should always be included as part of differential diagnosis in human patients presenting peripheral lung nodules who live in (or have traveled to) endemic regions.
